# Adolescents’ Addictive Phone Use: Associations with Eating Behaviors and Adiposity

**DOI:** 10.3390/ijerph17082861

**Published:** 2020-04-21

**Authors:** Sarah E. Domoff, Emma Q. Sutherland, Sonja Yokum, Ashley N. Gearhardt

**Affiliations:** 1 Department of Psychology, Central Michigan University, Mount Pleasant, MI 48859, USA; domof1se@cmich.edu; 2 School of Public Health, University of Michigan, Ann Arbor, MI 48109, USA; emmasuth@umich.edu; 3 Oregon Research Institute, Eugene, OR 97403, USA; sonjas@ori.org; 4 Department of Psychology, University of Michigan, Ann Arbor, MI 48109, USA

**Keywords:** phone, adolescents, addiction, BMI percentile, food addiction, emotional eating, impulsivity, emotion regulation

## Abstract

Concerns have been raised about excessive or “addictive” phone use among adolescents, and the impact that addictive phone use (APU) can have on adolescent development and health. Most research on the physical health correlates of smartphone use has been limited to sleep health, whereas other outcomes, such as eating behaviors and obesity risk have not received as much attention. To address this gap in the literature, we examined the association between APU and emotion regulation difficulties, impulsivity, maladaptive eating behaviors, and adiposity in a sample of 111 adolescents. We found that APU is associated with greater emotion regulation difficulties, dysregulated eating, restrained eating, food addiction, and higher percent body fat. Further, we found that emotion regulation difficulties mediated the association between APU and dysregulated eating, restrained eating, and food addiction. Findings suggest that addictive phone use may confer increased risk for obesogenic eating behaviors and food addiction via challenges in regulating emotions.

## 1. Introduction

Smartphone access and ownership continues to increase among adolescents in the United States. Indeed, a majority of youth (53%) own a smartphone by age 11—this increases to over 80% of youth by age 14 [1]. In addition to gaining access to smartphones at younger ages, shifts have also occurred in frequency of smartphone use. Social media applications (apps) are more frequently used by teens, with 38% of teens stating they use social media multiple times per hour, and 16% indicating they use social media nearly constantly [2].

Given the massive shift in adolescents’ consumption of social media and other apps on mobile devices, concern has been expressed regarding whether adolescents can become “addicted” to screens, particularly smartphones. Nearly half of parents in the United States report that their children are addicted to mobile devices [3]. Although the term “addiction” may be used colloquially to describe too much use, the construct of addictive phone use is conceptualized in the scientific literature as a behavioral addiction, wherein excessive use leads to dysfunction or impairment [4]. Similar to the measurement of specific types of screen media addiction (e.g., problematic media use in children: [5]; social media dependence: [6]), symptoms of addictive phone use are based on the DSM-5 criteria for Internet Gaming Disorder (IGD [7]), but applied to smartphone use [4]. The nine criteria include indicators of both excessive use and dysfunction related to use, such as tolerance (needing to use phone for longer amounts of time), withdrawal (i.e., feeling irritable when unable to use or access phone), use to manage/modulate mood, and psychosocial impairment due to use.

Most research on addictive phone use has been conducted in college student and other adult populations [8]. Although a growing body of research has examined addictive phone use in adolescents in Europe and Asia [9], very limited research has examined addictive phone use among adolescents in the United States. One recent exception described the development of the Addictive Patterns of Use measure (APU measure) and its association with academic functioning in US adolescents. Domoff, Foley, and Ferkel (2019) found that greater addictive phone use (using the aforementioned DSM-5 criteria) is associated with poorer academic functioning, over and above the amount of time spent using social media on school days [4]. This study suggests that how adolescents use smartphones, and not necessarily the time they spend on smartphones, confers risks to adolescent development.

Traditional screen time (i.e., time spent watching TV) has long been associated with physical health outcomes, such as obesity and poorer sleep, in children and adolescents [10]. A recent review on the literature on physical health correlates of mobile devices noted major gaps in our understanding of the impact of excessive smart phone use on the physical health of youth [11]. Although research has most consistently linked smartphone use with disrupted sleep and shorter sleep duration [12], we are unaware of research examining addictive phone use with other health concerns, such as problematic eating behaviors, food addiction, and adiposity. The limited research that has considered smartphone use and health risks examines overall time spent on smartphones [13,14,15], but not addictive phone use. Given the high rates of phone use and concerns about phone addiction, it is critical to clarify whether addictive phone use may contribute to problematic eating behaviors and adiposity in US adolescents.

Furthermore, consideration of the underlying mechanisms accounting for the relationship between addictive phone use and physical health should be explored to clarify potential targets for clinical intervention. In particular, theoretical and empirical support exists for the role of impulsivity and emotion regulation difficulties on problematic phone use (see the Billieux et al. (2015) pathway model for the emergence of problematic mobile phone use [16]). Specifically, Billieux et al. (2015) outline three pathways that lead to problematic phone use. Two of these pathways, the excessive reassurance pathway and the impulsive pathway, describe both emotion regulation challenges (i.e., emotional instability) and poor impulse control (i.e., lack of premeditation, low self-control, and ADHD symptoms), as risk factors for addictive patterns of phone use. Billieux et al., (2015)’s theoretical pathways have been supported in studies of adolescents in Turkey and South Korea [17]. Similar correlates have also been defined as likely contributors to maladaptive eating behaviors, food addiction, and obesity in adolescents [18,19]. As such, impulsivity and emotion regulation difficulties will be explored as potential mediators.

In summary, smartphone addiction and its impact on adolescent health is largely understudied [11]. This is a significant area of research as a vast majority of US teens have their own smartphones, and nearly half of US parents are concerned about their adolescents’ addiction to mobile devices [3]. Although countless studies have examined additive phone use and adolescent mental health [20], no studies have examined addictive phone use and obesity risk in US adolescents. The United States has a high rate of childhood obesity, with one in five adolescents meeting criteria for obesity [21]. To our knowledge, no research has considered whether smartphone addiction confers obesogenic risk among US adolescents. Given that obesity prevention is critical to the health of adolescents, examining whether and how addictive phone use may increase obesity risk fits within the priorities of the National Institute of Child Health and Human Development’s Strategic Plan [22]. Preliminary research is necessary to investigate obesity-related health correlates of addictive phone use.

To address the limitations of previous research and set the foundation for future clinical research, our study aims to: (1) examine differences by gender, race/ethnicity, and parental education in addictive phone use; (2) investigate whether adolescents who report greater addictive phone use have greater emotion regulation difficulties, impulsivity, dysregulated eating, restrained eating, food addiction, and adiposity (objectively measured by body mass index (BMI) percentile and percent body fat); and (3) explore whether emotion regulation difficulties and impulsivity mediate the associations between addictive phone use and eating behaviors and adiposity.

## 2. Materials and Methods

### 2.1. Participants

This study is drawn from a larger parent study (*N* = 193) examining adolescents’ neural responsiveness to fast food advertisements [23]. Administration of the Addictive Patterns of Use measure (APU measure) occurred part-way through the study, for a final *N* of 111 adolescents (see Table 1 for demographic characteristics). Data collection occurred between July 2015 and August 2017.

### 2.2. Procedure

Study procedures were approved by the Institutional Review Board at the University of Michigan (UM IRB board: HUM00095596). After obtaining parent/legal guardian informed consent and adolescent written assent, participants completed two visits to the lab. These visits consisted of survey completion, anthropometric measurement, and a functional magnetic resonance imaging paradigm (see Gearhardt et al., 2020, for additional information) [23]. Participants received compensation for their participation.

### 2.3. Measures

#### 2.3.1. Demographic Characteristics

Information about the participants’ demographics (e.g., age, gender, ethnicity, race, and highest education level of parent in the household) was provided. We categorized race/ethnicity as White or non-White, and parental education was categorized as college degree or higher or no college degree attained.

#### 2.3.2. Addictive Patterns of Use (APU) Scale

Participants were administered the APU scale [4], which consists of nine items assessing excessive use of Smartphones (α = 0.85). Sample items are as follows: “During the last year, how often have you felt restless or tense when you were unable to use your phone?” and “During the last year, how often have there been times when all you could think about was using your phone?” Participants responded to items on a 5-point Likert scale, ranging from 1 (Never) to 5 (Very often). Mean scores were calculated to provide a total score of addictive phone use, with higher scores indicating greater addictive phone use.

#### 2.3.3. Difficulties in Emotion Regulation Scale (DERS)

The DERS is a 36-item scale (α = 0.90) which assesses adolescents’ perceptions of difficulties in regulating emotions [24]. Sample items include, “I have difficulty making sense out of my feelings” and “When I’m upset, I have difficulty controlling my behaviors.” Adolescents respond on a Likert scale ranging from Almost Never (1) to Almost Always (5). Responses were summed to calculate a total score, with higher scores indicating greater difficulties in emotion regulation.

#### 2.3.4. Barratt Impulsiveness Scale-Brief (BIS-Brief)

The BIS-Brief is a shortened version of the BIS-11, a widely used measure of trait impulsivity [25]. The BIS-Brief consists of eight items (α = 0.76), including “I do things without thinking” and “I am self-controlled.” Participants respond to questions on a scale from rarely/never (1) to almost always/always (4). Responses are summed to provide a total score, with higher scores indicating greater trait impulsivity.

#### 2.3.5. Eating Behaviors

The Dutch Eating Behaviour Questionnaire (DEBQ) is a widely used self-report measure of three types of eating behaviors: restrained eating, emotional eating, and external eating [26]. Participants responded to items on a 5-point Likert scale from (1) never to (5) very often (33 items). Restrained eating is measured by 10 items (α = 0.93) such as “Do you try to eat less at mealtimes than you would like to eat?” The mean of these items was calculated to provide an overall restrained eating score. Emotional eating is measured by 13 items (α = 0.91), such as “Do you get the desire to eat when you are anxious, worried or tense?” and external eating is measured by responses to 10 items (α = 0.86), such as “If you see or smell something delicious, do you have a desire to eat it?” Given the high inter-correlation of emotional and external eating scales (*r* = 0.58, *p* < 0.01), the mean of the emotional and external eating items was calculated for a score of dysregulated eating.

#### 2.3.6. The Dimensional Yale Food Addiction Scale for Children 2.0 (dYFAS-C 2.0)

The dYFAS-C 2.0 [27] consists of 16 items (α = 0.91), adapted from the adult version of the Yale Food Addiction Scale 2.0 [28]. Items include: “When I started to eat certain foods, I found it hard to stop” and “When I cut down or stopped eating certain foods, I craved them a lot more.” Participants responded to questions on a 5-point Likert-scale, ranging from Never (0) to Always (4). The sum of items was calculated to provide a total score. Higher scores indicate higher levels of food addiction.

#### 2.3.7. Body Mass Index (BMI) Percentile

Objectively measured height and weight via standardized anthropometric measurements occurred, with participants instructed to wear light clothing. Height was measured using an O’Leary Acrylic Stadiometer (Ellard Instrumentation LLD, Monroe, Washington, USA) height (in centimeters, to the nearest tenth); weight was measured using a Detecto Portable Scale (Cardet, Webb City, Missouri, USA) (in kilograms, to the nearest tenth). Sex, age, height, and weight were used to calculate BMI percentiles [29].

#### 2.3.8. Percent Body Fat

After participants completed the standard anthropometric measurements, we also objectively measured their percent body fat (PBF) via InBody 570 scale (InBody USA, Cerritos, California, USA), with an 8-point Bioelectrical Impedance Analyzer (Frequencies 5, 50, 500 kHz). Consistent with the measurement of height and weight, participants were instructed to wear light clothing and to remove coats, shoes, and socks for PBF measurement.

### 2.4. Statistical Analyses

All variables used in this study were investigated for normality. Given that some of the variables were skewed, non-parametric analyses that do not require normal distributions were used. For Aim 1, Mann-Whitney tests were used to compare mean APU scores across demographic categories. For Aim 2, Spearman rho correlations were used to investigate the association of APU with the outcome variables. For Aim 3, Hayes’ PROCESS macro (Model 4) was used to explore whether emotion dysregulation and impulsivity mediated the associations examined in Aim 2 [30]. Due to identified sex differences in APU, sex was entered as a covariate in the mediation analyses.

## 3. Results

### 3.1. Participant Characteristics:

The mean age of the participants was 14.57 (*SD* = 1.08) years. The average BMI percentile was 78.59 (*SD* = 21.84) and the average percent body fat (PBF) was 26.84 (*SD* = 11.60). The sample was 44.1% male (*n* = 49); 71.2% identified as White (*n* = 79), followed by 17.1% identifying as Black/African American (n = 19; see Table 1 for additional characteristics of the sample).

### 3.2. Aim 1

Demographic differences in APU were examined by gender, race/ethnicity, and parent education level. Girls (*M* = 2.05, *SD* = 0.49) reported greater APU than boys (*M* = 1.55, *SD* = 0.60; *t* (109) = −4.69, *p* < 0.01). No significant differences emerged by race/ethnicity and parent education level (*p*’s > 0.05).

### 3.3. Aim 2

To investigate whether adolescents who report greater APU have greater emotion regulation difficulties, impulsivity, dysregulated eating, restrained eating, food addiction, and adiposity (as measured by BMI percentile and percent body fat), we conducted Spearman’s Rho correlations. We found significant associations between APU and emotion dysregulation, dysregulated eating, restrained eating, food addiction and percent body fat (see Table 2). APU was not significantly associated with impulsivity, and was thus not included in the exploratory mediation analyses. APU did not significantly associate with BMI percentile.

### 3.4. Aim 3

To explore whether emotion regulation difficulties mediated the associations between addictive phone use and maladaptive eating behaviors and adiposity, we used Hayes’ (2017) PROCESS macro (Model 4). We found that emotion regulation difficulties mediated the association between APU and dysregulated eating (see Table 3), restrained eating (see Table 4), and food addiction (see Table 5).

## 4. Discussion

A major aim of this study was to examine the physical health correlates of APU that are under-studied in the literature and that are major areas of public health concern in the United States (see Domoff, Borgen, Foley, & Maffett, 2019 [11] for a review). Specifically, we sought to investigate whether addictive phone use associated with objectively measured indicators of obesity (i.e., percent body fat and body mass index, BMI), as well as adolescents’ eating behaviors which have consistently been linked to obesity risk [31]. Consistent with prior research on non-mobile media (e.g., TV, computer, gaming [14]), we similarly found that addictive phone use associates with various obesogenic risk factors, including dysregulated eating, food addiction and percent body fat. We, however, did not find an association between addictive phone use and BMI percentile. It is possible that percent body fat might be a more accurate measure of adiposity. BMI percentile scores may be biased for individuals with elevated lean muscle mass.

We also sought to explore whether emotion regulation difficulties mediated the association of addictive phone use with dysregulated eating, restrained eating, food addiction, and adiposity. We found that emotion regulation difficulties mediated the association between addictive phone use and dysregulated eating, restrained eating, and food addiction, but not adiposity. It is possible that emotion regulation difficulties may be an underlying or common factor for both maladaptive eating behaviors (e.g., dysregulated eating, food addiction) and excessive phone use. As has recently been suggested [32], overlap in the experience of multiple addictions (including behavioral addiction) may indicate an underlying phenotype for addiction proneness. Future research may seek to test out other common factors across addictions, as has been conducted specific to screen-based behavioral addictions (e.g., [24]). Though preliminary, these findings suggest that clinicians assisting youth with emotion regulation challenges should assess for the presence of co-occurring addictions, such as phone addiction and food addiction.

Consistent with prior research in countries outside of the US [9,33], we found that girls had higher rates of addictive phone use and no differences emerged by race/ethnicity or parent education level. Given the social features of several popular smartphone apps (e.g., social media, texting or communication apps), the higher utilization of social media by girls [1], and the salience of peer relations in girls during adolescence, it is understandable that they would be at heightened risk for excessive phone use and dysfunction related to phone use. Although prior research on access to mobile devices and amount of screen time differs across various demographic factors [1], we examined addictive phone use and not hours of smartphone use. When prior research has examined problematic smartphone use, similar findings have emerged in adolescents in other Western countries [9].

### Limitations

There are limitations of the study that should be addressed in future research. First, our sample size was small; future research should seek to replicate our findings in a larger, more representative sample of adolescents. It is possible that, with a larger, more representative sample, impulsivity may significantly associate with addictive phone use, as has been found in research on young adults in the US [34]. We should also note that a larger sample size would be beneficial to conduct more sensitive analyses to understand how the associations may be moderated by demographic factors (e.g., race, ethnicity, gender). As has been found in prior research on problematic phone use (e.g., [9,33]), girls may be at greater risk for compulsive use. Additional research should consider gender and stage of development (early adolescent versus older adolescent/emerging adult) in future research. Additionally, the current cross-sectional study does not permit unambiguous conclusions regarding temporal precedence and the directions of effects. It is important to emphasize, however, that given this is the first study to examine the links between addictive phone use and obesity, and underlying mechanisms (i.e., emotion regulation) connecting the constructs, it would have been premature to carry out a longitudinal design as preliminary research was lacking. The current study has thus set the foundation for future research on physical health correlates of addictive phone use, which should utilize a longitudinal design, as some of the physical health outcomes (e.g., obesity) may not emerge until later in adolescence or early adulthood, and to demonstrate temporal precedence and direction of effects.

## 5. Conclusions

This study provides preliminary evidence demonstrating the physical health risks associated with addictive phone use in US adolescents. Further, we found that emotion regulation difficulties appear to be a mechanism through which youths’ addictive phone use may associate with dysregulated eating, restrained eating, and food addiction. On the other hand, it may also be possible that youth with greater emotion regulation concerns are at heightened risk for multiple addictions (e.g., food addiction, addictive phone use). Future research should seek to replicate our findings in a larger, more diverse sample of adolescents in order to inform clinical recommendations and intervention targets.

## Figures and Tables

**Table 1:** Sample Characteristics.

Characteristics	M (*SD*)/*n* (%)
Age	14.57 (1.08)
Gender	
Female	62 (55.9%)
Male	49 (44.1%)
Ethnicity	
Hispanic	9 (8.1%)
Race	
White	79 (71.2%)
Black	19 (17.1%)
Biracial	8 (7.2%)
Other	5 (4.5%)
Parent education level	
Less than high school or less	12 (10.8%)
High school diploma	7 (6.3%)
Some college courses	18 (16.2%)
Associate’s degree	10 (9.0%)
Bachelor’s degree	30 (27.0%)
Advanced degree	34 (30.6%)
BMI Percentile	78.59 (21.84)
Percent body fat	26.84 (11.60)
Dysregulated eating	1.97 (0.52)
Restrained eating	2.06 (0.87)
DERS	74.52 (17.37)
BIS-Brief	15.96 (3.66)
dYFAS-C 2.0	26.89 (8.89)
APU	1.83 (0.60)

Note: BMI—Body Mass Index; Dysregulated eating was measured with the external and emotional eating subscales of the Dutch Eating Behaviour Questionnaire (range 1–5); DERS—Difficulties in Emotion Regulation Scale (range: 36–180); BIS-Brief—Barratt Impulsiveness Scale, Brief version (range: 8–32); dYFAS-C 2.0—Dimensional Yale Food Addiction Scale for Children, 2.0 (range: 0–64); APU—Addictive Patterns of Use Scale (range: 1–5).

**Table 2:** Associations of Addictive Phone Use (APU) with Emotion Regulation Difficulties (DERS), Impulsivity (BIS-Brief), Eating Behaviors, Food Addiction (dYFAS-C 2.0), Body mass index (BMI) Percentile, and Percent Body Fat (PBF).

Variables	1	2	3	4	5	6	7	8
1. APU	-							
2. DERS	0.29 **	-						
3. BIS-Brief	0.18 ^+^	0.20 **	-					
4. Dysregulated eating	0.53 **	0.51 **	0.16 *	-				
5. Restrained eating	0.39 **	0.33 **	0.07	0.68 **	-			
6. dYFAS-C 2.0	0.49 **	0.35 **	0.07	0.56 **	0.39 **			
7. BMI percentile	0.08	0.10	0.00	0.25 **	0.43 **	0.32 **	-	
8. PBF	0.22 *	0.04	−0.04	0.27 **	0.37 **	0.40 **	0.76 **	-

Note: APU—Addictive Patterns of Use Scale; DERS—Difficulties in Emotion Regulation Scale; BIS-Brief—Barratt Impulsiveness Scale, Brief version; dYFAS-C 2.0—Dimensional Yale Food Addiction Scale for Children, 2.0; BMI—Body Mass Index; PBF—Percent Body Fat; **. *p* < 0.01; *. *p* < 0.05.; +. *p* < 0.10.

**Table 3:** Emotion Regulation Difficulties (DERS) as a Mediator of the Relation Between Addictive Phone Use (APU) and Dysregulated Eating.

Model	*B*	*SE*	*t*	*p,* Cis	*F*	*R^2^*
Model summary				0.00	*F* (3, 107) *=* 24.47	0.41
Direct effects						
APU	0.33	0.07	4.43	0.00		
DERS	0.01	0.00	4.480	0.00		
Sex	0.11	0.08	1.32	0.19		
Indirect effect						
DERS	0.10	0.03 ^a^		(0.04, 0.17) ^b^		
Completely Standardized Indirect Effect						
DERS	0.11	0.04 ^a^		(0.05, 0.19) ^b^		

Note: APU—Addictive Patterns of Use Scale; DERS—Difficulties in Emotion Regulation Scale. Estimates are unstandardized. ^a^ Bootstrap-derived estimate of standard error of indirect effect, 10,000 bootstrapped samples; ^b^ 95% lower and upper level confidence intervals.

**Table 4:** Emotion Regulation Difficulties (DERS) as a Mediator of the Relation Between Addictive Phone Use (APU) and Restrained Eating.

Model	*B*	*SE*	*t*	*p*, CIs	*F*	*R^2^*
Model summary				0.00	*F* (3, 107) *=* 11.69	0.25
Direct effects						
APU	0.45	0.14	3.21	0.00		
DERS	0.01	0.00	3.05	0.00		
Sex	0.12	0.16	0.71	0.48		
Indirect effect						
DERS	0.12	0.05 ^a^		(0.03, 0.24) ^b^		
Completely Standardized Indirect Effect						
DERS	0.09	0.04 ^a^		(0.02, 0.16) ^b^		

Note: APU—Addictive Patterns of Use Scale; DERS—Difficulties in Emotion Regulation Scale. Estimates are unstandardized. ^a^ Bootstrap-derived estimate of standard error of indirect effect, 10,000 bootstrapped samples; ^b^ 95% lower and upper level confidence intervals.

**Table 5:** Emotion Regulation Difficulties (DERS) as a Mediator of the Relation Between Addictive Phone Use (APU) and Food Addiction.

Model	*B*	*SE*	*t*	*p,* CIs	*F*	*R^2^*
Model summary				0.00	*F* (3, 86) *=* 11.25	0.28
Direct effects						
APU	3.97	1.55	2.57	0.01		
DERS	0.13	0.05	2.58	0.01		
Sex	3.29	1.80	1.83	0.07		
Indirect effect						
DERS	1.33	0.62 ^a^		(0.18, 2.64) ^b^		
Completely Standardized Indirect Effect						
DERS	0.09	0.04 ^a^		(0.01, 0.18) ^b^		

Note: APU—Addictive Patterns of Use Scale; DERS—Difficulties in Emotion Regulation Scale. Estimates are unstandardized. ^a^ Bootstrap-derived estimate of standard error of indirect effect, 10,000 bootstrapped samples; ^b^ 95% lower and upper level confidence intervals.
